# Freeze the Disease: Advances the Therapy for Barrett’s Esophagus and Esophageal Adenocarcinoma

**DOI:** 10.3390/cancers18010059

**Published:** 2025-12-24

**Authors:** Ted G. Xiao, Shree Atul Patel, Nishita Sunkara, Virendra Joshi

**Affiliations:** 1 Department of Digestive Diseases, Emory University, Atlanta, GA 30322, USA; ted.xiao@emory.edu; 2 Department of Internal Medicine, Emory University, Atlanta, GA 30322, USA; shree.patel@emory.edu (S.A.P.); nishita.sunkara@emory.edu (N.S.)

**Keywords:** cryotherapy, Barrett’s esophagus, radiofrequency ablation, strictures, cryo-immunology

## Abstract

Cryotherapy involves flash freezing of tissue and removing unwanted tissue. Mechanism of injury is causing cell membrane rupture by rapid multiple freeze–thaw cycles, while reserving tissue architecture and the collagen matrix. This promotes favorable wound healing. In recent years, it has gained increasing attention as a treatment option for upper gastrointestinal diseases (Barrett’s Esophagus and early cancer). Currently, two FDA-approved delivery methods are available in the GI tract: Cryoballoon and spray cryotherapy, which will be discussed. In this review, we also propose to examine the expanding role of cryotherapy in gastrointestinal practice, drawing from both clinical studies and illustrative vignettes. In addition, we will highlight its established role in eradicating Barrett’s with low and high-grade dysplasia and compare its outcomes and safety profile with radiofrequency ablation (RFA). We will also discuss the application and safety of spray cryotherapy in the palliation of malignant esophageal strictures when compared with Esophageal stent placement. Cryotherapy may have immunological potential, and it may shrink both primary and metastatic diseases. Ongoing research in this field of Cryo-immunology will be highlighted. Beyond esophageal neoplasia, cryotherapy is increasingly utilized in other upper gastrointestinal precancerous conditions. Through this synthesis, our goal is to provide a timely and comprehensive overview of advancements in cryotherapy and its potential to reshape novel therapeutic approaches in upper gastrointestinal cancers. Finally, we highlight the evolution of a novel platform using nitrous oxide delivered by a handheld device, a contact balloon, and a small replaceable cartridge. This approach may make delivery of cryogen application favorable and a first-line approach in the management of Barrett’s esophagus and early cancer. In addition, Cryoballoon therapy for dysphagia palliation for malignant esophageal strictures may become a preferred approach as more data evolves.

## Simple Summary

Cryotherapy is a medical technique that uses extreme cold to “flash freeze” and remove unhealthy tissue in the digestive tract. Research is being suggested to prove it is a safer, more effective alternative for treating early-stage cancers and Barrett’s Esophagus compared to traditional “burning” methods. By destroying bad cells while keeping the body’s natural structural “scaffolding” intact, it promotes better healing. For the research community, the most exciting impact is Cryo-immunology, where freezing a tumor may “wake up” the immune system to recognize and attack cancer cells throughout the body. Additionally, studying new handheld delivery devices aims to establish cryotherapy as a standard, first-line treatment. By proving it can effectively clear blockages and improve a patient’s ability to swallow, this research could shift medical practice toward simpler freezing methods that significantly improve quality of life.

## 1. Introduction

Upper GI malignancies remain a leading cause of cancer-related morbidity and mortality worldwide. Chronic gastroesophageal reflux disease leads to the development of Barrett’s metaplasia, the main precursor lesion for esophageal adenocarcinoma. This subtype has continued to increase in the Western world, while the squamous subtype is decreasing. Endoscopic screening and surveillance strategies have not significantly impacted the incidence and prevalence of esophageal neoplasia in the Western world [1,2]. Early detection and ablation of dysplastic Barrett’s esophagus remain the standard of care [3,4]. This has led to the development of various ablative and mucosal resection techniques, such as photodynamic therapy, argon plasma coagulation, radiofrequency ablation, endoscopic mucosal resection, and endoscopic mucosal dissection [3]. These novel techniques, alone or in combination, have since been applied in managing Barrett’s high-grade dysplasia (HGD) and early mucosal cancer of the esophagus [4]. However, they also have limitations, issues with ablation, thermal injury, strictures, and incomplete eradication. This gap has spurred further exploration of other modalities, including mucosal resection and, more recently, cryotherapy—a novel treatment approach that promotes favorable wound healing through rapid multiple freeze–thaw cycles at temperatures below 50 degrees Celsius. Endoscopic cryotherapy has shown early therapeutic success in the treatment of Barrett’s esophagus. Since then, it has gained increasing relevance in managing various other gastrointestinal pathologies. The purpose of this review is to synthesize current evidence on cryotherapy applications in malignant and pre-malignant upper GI conditions, highlighting its potential to reshape therapeutic approaches in upper gastrointestinal malignancy. This review, for the first time, highlights the evolution of a new cryogen, nitrous oxide, delivered via a handheld device into a contact balloon and a small replaceable cartridge. This targeted approach and ease of application may make this platform a first-line approach for the management of Barrett’s esophagus and early cancer. Moreover, CBA for dysphagia palliation for malignant esophageal strictures may also become a favored approach in the future as more data evolves versus placing esophageal stents. Cryotherapy, in addition to its local effects of cellular necrosis and apoptosis, may induce systemic immunological effects that may work synergistically with immunotherapy in esophageal malignancy [5,6,7,8,9].

## 2. Evolution of Cryotherapy

Cold treatment has evolved considerably in the past 200 years, transitioning from broad, nonspecific uses such as hydrotherapy to the targeted, focal tissue destruction seen in modern cryosurgery. The therapeutic application of freezing temperatures began in England, where James Arnott, an English physician, published his work on iced salt solutions between 1819 and 1879 [10].

These early solutions were applied to freeze advanced cancers in accessible sites, producing reductions in tumor size and cancer-associated pain. At the time, however, cryosurgery remained a relatively minor technique, considered suitable only for accessible cutaneous and mucosal lesions. Over subsequent decades, renewed interest in cryosurgery spurred the development of methods adaptable to a wide range of clinical applications across multiple medical specialties [11,12]. Today, cryotherapy offers a uniquely minimally invasive therapeutic option, ideal for patients unfit for surgery.

## 3. Mechanism of Injury

**Figure 1:**
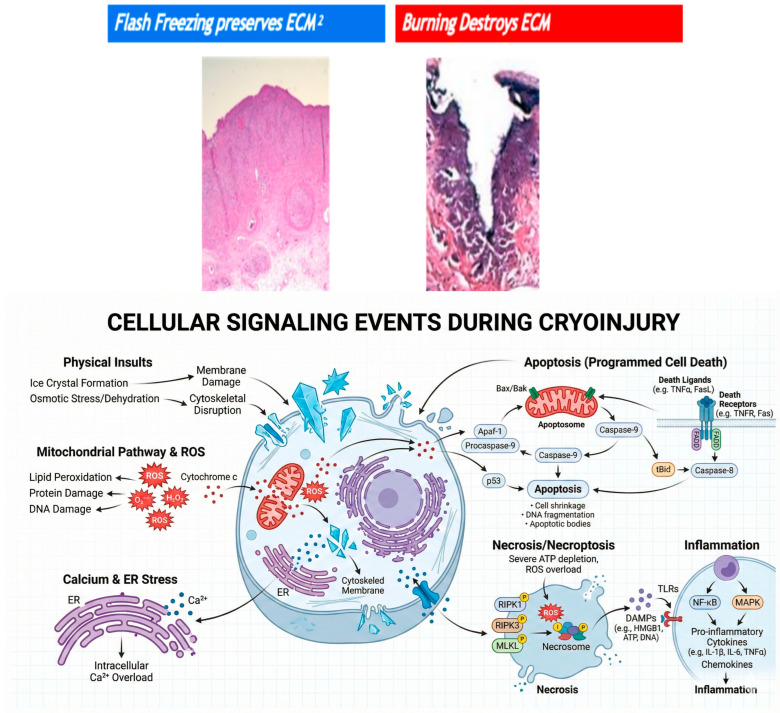
Comparison of tissue effects from cryotherapy versus thermal ablation. Flash-freezing preserves the extracellular matrix, whereas heat-based ablation causes deep stromal injury and ECM destruction. These images are from biopsies taken post-cryotherapy immediately, demonstrating preservation of stroma and intact histology [5,18]. Schematic diagram of a complex cellular signaling pathway during cryoinjury.

**Figure 2:**
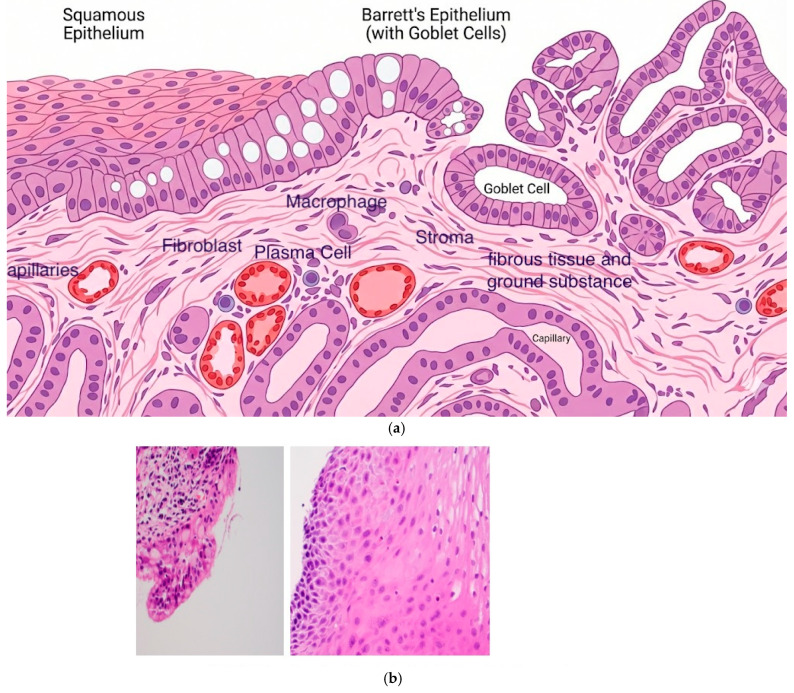
(**a**) Schematic representation of the extracellular matrix and associated cellular components, illustrating the structural scaffold preserved during cryotherapy [16]. (**b**) Barrett’s without dysplasia-Post Ablation with balloon cryotherapy. Demonstrates intact histology, Biopsies taken within minutes post ablation.

Cryotherapy offers a fundamentally different mechanism of action in the eradication of malignancy. The extreme cold, down to −196 °C, causes intracellular ice crystals to form, expand, and burst, destroying cells with higher fluid content (Figure 1). The subsequent slow thaw provides larger ice crystals, inducing both apoptosis and vascular stasis [13,14,15]. Given that the extracellular matrix is mostly collagen and has a low fluid content, it is preferentially protected against the flash freeze (Figure 2). This can then serve as a scaffold for the growth of new healthy tissue [16]. Additionally, the microcirculatory failure that follows thawing deprives the cells of oxygenation, resulting in additional ischemic death [17]. This process has been shown to yield similar levels of cell death as other heat-based modalities, such as radiofrequency ablation or argon plasma coagulation; however, with preservation of the underlying tissue architecture and extracellular matrix [5].

## 4. Types of Endoscopic Cryotherapy Methods

**Figure 3:**
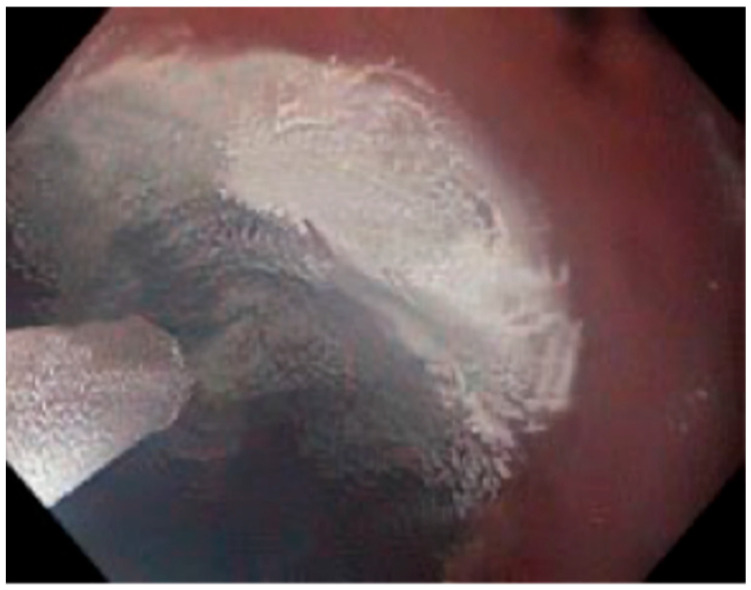
Endoscopic view of liquid nitrogen spray cryotherapy being applied to esophageal mucosa using a through-the-scope spray catheter. Images supplied courtesy of Dr. Virendra Joshi.

There are currently two major FDA-approved methods of cryotherapy delivery: spray cryotherapy and Cry balloon ablation (CBA). In spray Cryotherapy, liquid nitrogen is delivered through a flexible spray catheter passed through the endoscope, applying cryogen directly onto the target tissue (Figure 3). After application, the applied liquid nitrogen locally expands into a gaseous form. For this reason, a decompression tube is also necessary to prevent overinflation of the area [19]. The ablation dose is determined by the duration of nitrogen application, followed by a controlled period of thawing. Multiple freeze–thaw cycles are subsequently applied to achieve the effect.

**Figure 4:**
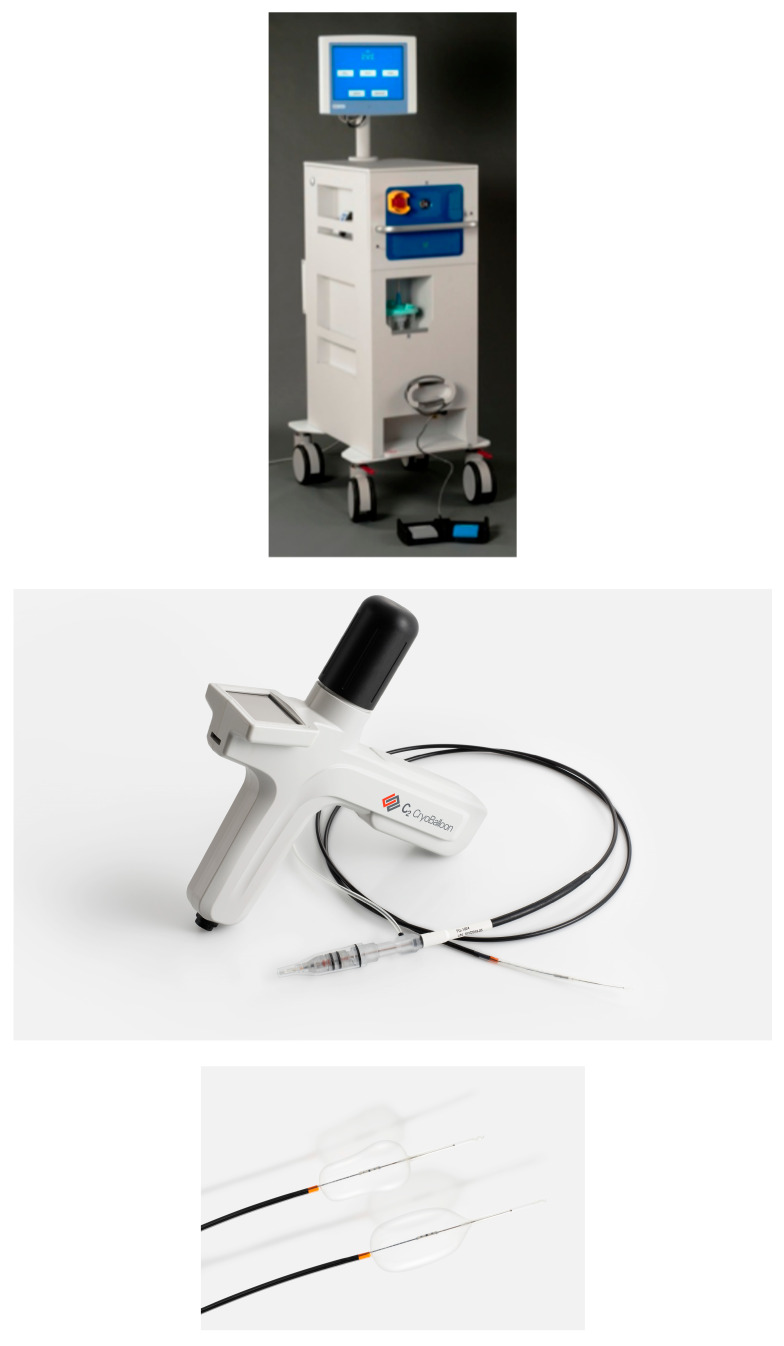
Image of: Console for LNCST (trufreeze^R^), Steris Healthcare. Cryoballoon handheld platform. Images supplied courtesy of Virendra Joshi, MD.

**Figure 5:**
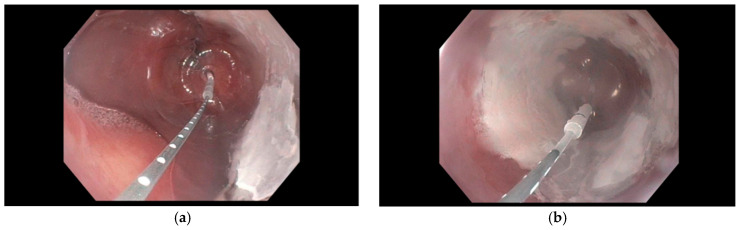
Cryoballoon ablation (CBA) catheter (**a**) 180° CBA application targeting a segment of esophageal mucosa; (**b**) Circumferential 360° CBA application. Images supplied courtesy of Dr. Virendra Joshi.

In contrast, CBA (Figure 4) uses an inflatable balloon cooled by nitrous oxide. This system also employs a through-the-scope catheter with an attached balloon operated with a foot pedal and hand-held controller. Once positioned at the site of interest, the balloon is inflated to match the diameter of the esophageal lumen. Pressurized liquid nitrogen is then released into the interior of the balloon through a diffuser, cooling both the balloon and the surrounding tissue (Figure 5). This process uniquely does not require direct contact of nitrous oxide with the target tissue. Because the gas remains contained within the balloon and is vented back through the catheter into an external sponge, no decompression tube is needed [20]. Additionally, CBA requires only a single freeze–thaw, further simplifying the ablation process.

Currently, spray cryotherapy is the most used modality. However, this technique is limited by several drawbacks, including the need for precise sizing of catheters, multiple deployment steps, imprecise dosing, and mandatory gas-venting, as outlined above. Other challenges that limit widespread adaptation include a bulky platform and procedure time. The newer Cryo-balloon Focal and the newly FDA-approved 180 Ablation catheter System may help overcome these limitations and are steadily gaining broader clinical adoption in expert hands.

## 5. Depth of Injury and Buried Glands

**Figure 6:**
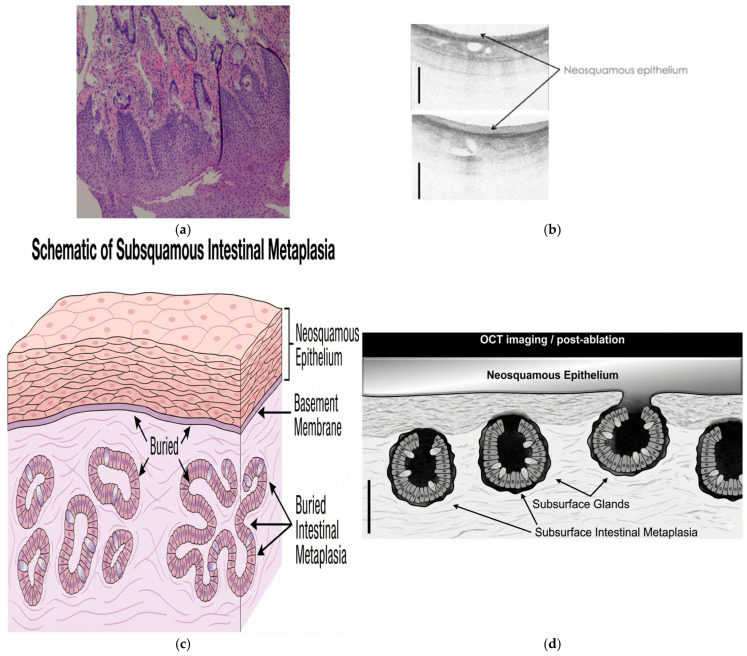
(**a**) Subsurface buried glands within the lamina propria [25].(**b**) Neo-squamous epithelium visualized on cross-sectional optical coherence tomography (OCT), demonstrating restoration of layered mucosal architecture following cryotherapy and submucosal glands. Buried Glands have been demonstrated with this system [22]. (**c**) Schematic Illustration of Subsquamous Metaplasia. (**d**) Shows buried Barrett’s Metaplasia under neo-squamous epithelium, these have been suggested as a source of recurrence of neoplasia post ablative therapy. Images: courtesy: Virendra Joshi, MD.

The significance of buried metaplasia or sub-squamous metaplasia remains unclear [21] (Figure 6). The presence of subsurface Barrett’s glands before and after radiofrequency ablation has been a source of concern (Figure 6). These glands are not exposed to elements that transform them to cancer; therefore, the risk should be low. They are often found at the edge of squamous and columnar epithelium at the GE junction post-ablation and under neo-squamous epithelium and have been attributed to recurrent disease and poor response. Crossover therapy to Cryoablation seems to improve response [21,22,23]. The thickness of Barrett’s or buried glands in the lamina propria could potentially explain failed thermal therapy. Though the cells in these glands are less proliferative, the exact risk of transformation to neoplasia needs prospective follow-up post-therapy [24,25].

## 6. Safety and Efficacy of Cryotherapy

The earliest clinical applications of cryotherapy in the gastrointestinal (GI) tract focused on the eradication of Barrett’s esophagus (BE). Over the past decade, many studies helped firmly establish its safety and efficacy (Table 1). Liquid nitrogen spray cryotherapy (LNSCT) has been the most widely studied methodology of cryotherapy for BE. In one of the earliest single-center studies, Gosain et al. treated 32 patients with BE with high-grade dysplasia (BE-HGD) using LNSCT every 8 weeks (about 2 months) until histologic eradication was achieved. After at least two years of surveillance, complete eradication of both HGD (CE-HGD) and intestinal metaplasia (CE-IM) was observed in most patients. The most common adverse event was stricture, which developed in 9% but was all successfully dilated [25,26]. This underscored cryotherapy’s efficacy and favorable safety profile.

Building on these initial findings, subsequent studies have demonstrated that the benefits of cryotherapy extend well beyond short-term eradication. Long-term outcomes have been equally encouraging. Ramay et al. demonstrated durable eradication in a cohort of 50 and then 40 patients over three- and then five-year follow-ups. Initial CE-HGD, CE-dysplasia (CE-D), and CE-IM rates were 98%, 90%, and 60%, respectively, with sustained eradication in 93% with HGD and 75% with IM after five years. Recurrence rates per person-year were only 1.4% for HGD/esophageal adenocarcinoma and 12.2% for IM, highlighting the lasting efficacy of cryotherapy [27].

These promising single- and multicenter results have since been substantiated by pooled analyses. A comprehensive systematic review by Chandan et al., which included 707 patients across 14 studies, further corroborated these outcomes. The pooled rates of CE-HGD, CE-D, and CE-IM were 90.3%, 80.8%, and 55.8%, respectively. Importantly, the rates of stricture and perforation were comparable to those reported for radiofrequency ablation (RFA) [28]. This reinforced that cryotherapy offers a similar safety margin as the current standard of care.

Beyond liquid nitrogen, other cryotherapy techniques have also demonstrated comparable safety and efficacy in the management of Barrett’s esophagus. Carbon dioxide (CO_2_) cryotherapy has been shown to achieve similar therapeutic outcomes. In a retrospective study of 64 patients, Canto et al. reported that all treatment-naïve cases achieved complete response in one year, while 91% of those treated in the rescue setting also reached complete response. Long-term follow-up demonstrated durable neoplastic eradication (87%) with minimal complications—only 3% of patients experienced serious adverse events, and no new strictures were observed in the cohort [29].

Further diversification of cryotherapy technology has led to the development of balloon-based systems, which provide controlled and targeted delivery of cryogen to diseased mucosa. Evidence from a meta-analysis of seven studies totaling 272 patients by Westerveld et al. demonstrated pooled CE-D and CE-IM rates of 93.8% and 85.8%, respectively. The overall adverse event rate was 12.5%, most commonly mild strictures in 6% of the cohort [30]. Similarly, multimodal treatment strategies that incorporate cryotherapy with radiofrequency ablation (RFA) or endoscopic mucosal resection (EMR) have achieved high rates of histologic eradication. In a retrospective cohort, Kaul et al. observed CE-IM in 75% and CE-D in 98% of 52 patients treated with combination approaches [31]. The durability of cryoballoon ablation is exemplified by Dbouk et al., who followed 59 treatment-naïve patients with dysplastic Barrett’s esophagus (BE) for a median of 54.3 months. At 1-year, complete eradication of dysplasia (CE-D) and intestinal metaplasia (CE-IM) was achieved in 94.6% and 75% of patients, respectively. Among those who achieved eradication, CE-D and CE-IM were maintained at 2 and 3 years in 100% and approximately 98% of cases, with cumulative recurrence rates of only 1.9% for dysplasia and 14.6% for intestinal metaplasia [32].

Taken together, these findings demonstrate that cryotherapy (i.e., liquid nitrogen, CO_2_, or balloon-based systems) is a highly effective and well-tolerated modality for BE eradication. Adverse events are uncommon and generally mild, confirming its favorable safety profile across a broad range of patient populations and settings.

**Table 1:** Studies on the efficacy and safety of cryotherapy for Barrett’s esophagus.

Studies	Type of Study	Patients Included	Efficacy	Safety & Recurrence	Median Follow-Up
Gosain et al. [26]	Observational	32 BE-HGD	32/32 CE-HGD 27/32 CE-IM	AE: 3 strictures	2 years
Canto et al. [29]	Retrospective Single center	20 treatment naïve 44 rescue treatment	Year 1: 10/13 CE-EAC 60/64 CE-D 35/64 CE-IM Long-term CE for neoplasia: 56/64	AE: 2 serious events 4 post cryotherapy pain Recurrence: 20/64 IM	4 years
Ghorbani et al. [33]	Prospective Multicenter	23 LGD 57 HGD	LGD: 91% CE-D 61% CE-IM HGD: 81% CE-D 65% CE-IM Short segment BE: 97% CE-D, 77% CE-IM	AE: 1 stricture 1 GI bleed	2 years
Ramay et al. [34]		50 BE-HGD or EAC	Year 3: 48/50 CE-HGD 47/50 CE-D 41/50 CE-IM Year 5: 37/40 CE-HGD 35/40 CE-D 30/40 CE-IM	AE: None Recurrence: 12.2% IM 4% Dysplasia 1.4% HGD/EAC	5 years
Kaul et al. [31]	Retrospective Single center	57 long-segment BE	39/52 CE-IM 51/52 CE-D	AE: 1 GI bleed 1 esophageal micro perforation Recurrence: 3/39 IM 4/39 Dysplasia 2/39 HGD	4.8 years
Dbouk et al. [32]	Prospective Single center	59 BE with LGD, HGD, or EAC	Year 1: 53/56 CE-D 42/56 CE-IM Year 3: 45/45 CE-D 40/41 CE-IM	AE: 5 stricture 1 GI bleed Recurrence: 14.6% IM 1.4% Dysplasia	4.5 years
Eluri et al. [35]	Prospective Multicenter	138 BE with LGD, HGD, and EAC	Year 2: 66% CE-IM 84% CE-D Year 3: 67% CE-IM 92% CE-D	AE: 5.5% stricture 0.7% perforation Recurrence: 8.8% IM	2.5 years

## 7. Comparison of Cryotherapy and Radiofrequency Ablation

Having demonstrated that cryotherapy is a highly effective and safe treatment option for patients with Barrett’s esophagus, investigators looked at cryotherapy performance in direct comparison with RFA as a primary treatment modality (Table 2). Direct comparisons between cryotherapy and RFA provide important insights into their relative efficacy and safety. In a retrospective cohort, Thota et al. compared outcomes among 81 cryotherapy and 73 RFA patients. It reported that RFA achieved higher CE-IM rates compared to cryotherapy (odds ratio 2.9); however, CE-D rates were similar between groups [36]. Similarly, a systematic review by Mohan et al. found that while CE-D outcomes were comparable, cryotherapy was associated with slightly lower CE-IM rates [37].

More recent evidence has challenged these early findings regarding the efficacy of cryotherapy in direct comparison to RFA. A multicenter study by Fasullo et al. found no significant differences in CE-D or CE-IM between 62 LNSCT and 100 RFA patients. It did report that cryotherapy required approximately one additional session to achieve complete eradication compared to RFA [38]. A 2024 meta-analysis by Papaefthymiou et al. pooled data from 23 studies that included 1604 patients. This study confirmed that CE-D and CE-IM rates for cryotherapy were 84.2% and 64.1%, which were statistically equivalent to those found in the RFA cohort. Recurrence and stricture rates were 8.3% and 6.5%, which were also comparable [39]. These studies taken together support the notion that cryotherapy offers efficacy and safety such as RFA. A large recent retrospective cohort study, Sachdeva et al., found a higher recurrence risk after RFA on multivariate analysis based on a 681-patient cohort [40].

Beyond retrospective and comparative studies, prospective data now provide dedicated support for cryotherapy as a primary therapy. The first prospective study evaluated nitrous oxide–based cryoballoon ablation in 41 patients with BE harboring low-grade dysplasia, high-grade dysplasia, or intramucosal carcinoma over 12 months. CE-D and CE-IM rates were 95% and 88%, respectively, after a median of three procedures per patient. Adverse events were limited to a single clinically insignificant upper gastrointestinal bleed in a patient on aspirin and strictures in four patients; median pain scores were zero on day 1 post-procedure [41]. A subsequent large prospective registry pooled 138 patients treated with liquid nitrogen spray cryotherapy across multiple community and academic sites between 2013 and 2022. At three years, CE-D and CE-IM rates were 92% and 67%, respectively. Longer BE segments (HR 0.9) and prior RFA treatment (HR 0.39) were associated with lower CE-IM rates. Recurrence occurred in 8.8%, with strictures and perforations in 5.5% and 0.7%, respectively [35]. These prospective studies provide high-quality evidence that cryotherapy is effective and safe as a first-line intervention, with outcomes approaching those historically reported with RFA.

Systematic reviews and meta-analyses provide additional evidence supporting cryotherapy as an effective primary treatment modality. Hamade et al. pooled data from six studies encompassing 282 patients and reported CE-IM and CE-neoplasia rates of 69.4% and 97.9%, respectively. Although 7% had persistent dysplasia and 4% progressed to cancer, overall eradication and recurrence rates (19.1 and 10.4 per 100 patient-years) were comparable to those achieved with RFA therapy [42]. A larger meta-analysis by Tariq et al. that included 14 studies and 405 patients further strengthened this evidence base. The pooled CE-IM and CE-D rates were 71.6% and 91.3%, with an adverse event rate of 12.2% [43]. These findings showed that cryotherapy performs at a comparable level to the current first-line therapy with a similar efficacy and safety profile. Thus, this suggests that cryotherapy can achieve robust eradication outcomes as a first-line intervention with an acceptable safety profile.

Cryotherapy provides efficacy and safety comparable to RFA while offering several procedural advantages. Although RFA remains the more established therapy for Barrett’s esophagus, cryotherapy is associated with lower post-procedural pain, reduced stricture risk, and greater flexibility in treating irregular or scarred mucosa. Supporting this, van Munster et al. demonstrated that focal cryoballoon ablation achieved a similar short-term treatment response to RFA for dysplastic BE but was associated with significantly lower pain scores, shorter duration of pain, and reduced analgesic use [44]. These benefits reinforce the utility of cryotherapy not only as salvage therapy but also as a potential first-line treatment option. Comparative studies have shown cryotherapy to be noninferior to RFA therapy and particularly suitable for patients with longer BE segments, fibrotic mucosa, or higher risk for thermal injury. Recent consensus recommendations by Shah et al. in The American Journal of Gastroenterology further provide guidance on indications, treatment intervals, and discontinuation criteria, helping to standardize its clinical application [45].

**Table 2:** Comparative studies for cryotherapy versus radiofrequency ablation.

Studies	Type of Study	No. Patients Included/Type of Therapy	Efficacy	Safety
Van Munster et al. [44]	Prospective 2-centers	20 Balloon cryotherapy 26 RFA	BE regression: Cryo = 88% RFA = 90% *p* = 0.62	Peak pain: cryo/RFA = 2/4 days Pain duration: cryo/RFA = 2/4 days Analgesic usage: cryo/RFA = 2/4 days All *P* < 0.01
Thota et al. [36]	Retrospective Single Center Heterogenous	81 cryotherapy 73 RFA	CE-IM: Cryo/RFA = 41%/67% CE-LGD Cryo/RFA = 79%/88% CE-HGD: Cryo/RFA = 88%/88% Progression: Cryo/RFA = 13%/13% Comparble response between RFA/LNSCT	Not reported
Canto et al. [29]	Prospective clinical trial	22 treatment naïve 19 previous RFA Treatment: Co2—Cryotherapy	CE-IM: 88% CE-D: 95% CE-HGD 95% CE-EAC 80%	4 strictures 1 GI bleed (aspirin)
Fasullo et al. [38]	Retrospective Multicenter	62 cryotherapy (LNSCT) 100 RFA	CE-D: LNSCT vs. RFA = 71% vs. 81%, *p* = 0.14 CE-IM: LNSCT vs. RFA = 66% vs. 64%, *p* = 0.78	No significant adverse event reported
Sachdeva et al. [40]	Retrospective 2-centers	71 cryoballoon 610 RFA Compared: Propensity matched 54 patients	Comparable Dysplastic recurrence rate Between CBA/RFA Baseline Barrett’s segment length independent risk factor for Dysplastic recurrence and all recurrence.	No significant adverse event reported

## 8. Cryotherapy as a Salvage Therapy in Refractory Barrett’s Esophagus

Cryotherapy has proven to be highly effective as a salvage treatment for patients with BE refractory to RFA or EMR. Early evidece supporting this role came from Halsey et al., who achieved a 92% complete response rate after repeat cryotherapy in 36 patients who previously failed RFA therapy [46]. These findings provided the first indication that cryotherapy could achieve meaningful histologic eradication even in persistent BE that failed first-line therapy.

Building upon these initial observations, subsequent investigations reinforced the efficacy and safety of cryotherapy in patients with BE refractory to RFA therapy. Sengupta et al. demonstrated CE-D in 75% of 16 RFA-refractory patients, with only mild strictures that responded readily to dilation [47]. Similarly, Trindade et al. reported CE-D and CE-IM rates of 72% and 50%, in 18 RFA refractory patients. These outcomes approached those achieved in treatment-naïve cohorts. Again, there were no procedure-related adverse events [48].

A meta-analysis by Visrodia et al. provided broader validation for cryotherapy as salvage therapy. This analysis encompassed 11 studies and 148 patients. It found pooled CE-D and CE-IM rates of 76% and 46%, with an overall adverse event rate of just 6.7% [49]. Spiceland et al. subsequently corroborated these results in a multicenter cohort of 46 refractory cases, reporting CE-D and CE-IM rates of 83% and 46% after a median of two cryotherapy sessions [50].

More recently, contemporary prospective data have confirmed these favorable outcomes in real-world practice. In a cohort of patients with persistent BE after failed RFA therapy, Genere et al. demonstrated CE-IM and CE-D rates of 83% and 96%. These results are comparable to repeating RFA treatment but with significantly fewer strictures in the cryotherapy group [27].

Taken together, this accumulation of evidence establishes cryotherapy as a consistently effective and safe salvage modality for BE refractory to standard therapies. Its non-contact mechanism, minimal tissue trauma, and favorable safety profile make it particularly well-suited for previously treated or fibrotic mucosa, providing an important therapeutic option in complex or recurrent disease.

## 9. Cryotherapy in the Palliative Management of Malignancy

**Figure 7:**
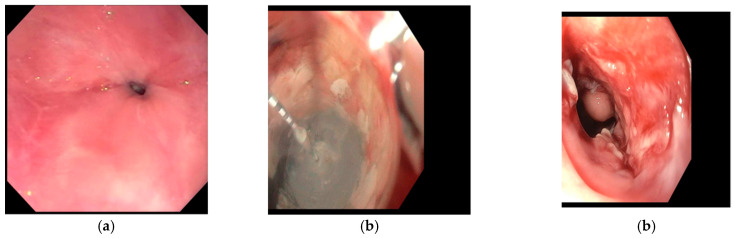
Endoscopic management of malignant esophageal stenosis using cryotherapy. (**a**) Malignant esophageal stricture causing near-complete luminal obstruction; (**b**) Application of liquid nitrogen spray cryotherapy across the malignant narrowing; (**c**) post-treatment appearance demonstrating opening of lumen following cryoablation. Images supplied courtesy of Dr. Virendra Joshi.

Recently, given its minimally invasive nature and relative efficacy, there has been growing interest in the use of cryotherapy as a tool in the palliative management of malignancy [51]. Cryotherapy has been increasingly adopted as a salvage option for the management of dysphagia in the setting of esophageal malignancy (Figure 7). In patients who are not candidates for esophagectomy, dysphagia can be profoundly debilitating and can significantly diminish quality of life.

Current first-line palliative strategies, such as radiation therapy and esophageal stent placement, are effective but carry substantial risks of adverse events. Complications from these procedures range from chest pain, reflux symptoms, tumor or tissue overgrowth, bleeding, perforation, and potential fistula formation [52,53]. Moreover, studies assessing the impact of stent placement on overall quality of life have shown mixed results [54]. These limitations have prompted increased interest in cryotherapy as an alternative option for palliation of dysphagia for these patients.

A retrospective, multicenter, case series of 49 inoperable esophageal cancer patients undergoing palliative endoscopic cryotherapy demonstrated a statistically significant improvement in dysphagia symptoms [55]. More notably, in a prospective multicenter cohort study, Kachaamy et al. found that the use of cryotherapy in combination with systemic chemotherapy not only improved dysphagia but also enhanced quality of life and reduced reflux symptoms [53]. This improvement in reflux symptoms can be considered a major advantage for cryotherapy over stenting. This suggests cryotherapy potentially has a role as the preferred treatment for palliation of dysphagia. Although existing studies are small, early evidence suggests that cryotherapy is a promising, safe, and effective treatment option for this patient population [51].

## 10. Cryo-Immunology

When diving into cryotherapy, it is critical to understand the mechanisms beyond freezing tumor cells when evaluating its utility and efficacy. Cryotherapy demonstrates a new phenomenon—cryo-immunology, the immune-mediated effects induced by cryotherapy itself.

Cryotherapy induces tumor cell death through controlled freeze–thaw cycles. In performing so, this therapy also exposes intact tumor antigens to the patient’s immune system and stimulates the release of pro-inflammatory signals (including TNF-alpha, IL-1, and IFN-γ). This elicits an anti-tumor immunological response from the patient’s innate and adaptive immune system, promoting recruitment of dendritic cells and T lymphocytes. Cryotherapy has been observed to shrink tumor burden in both primary and metastatic diseases present in the esophagus and the esophageal-gastric junction via tumor necrosis and apoptosis [19].

Significantly, cryotherapy differs from radiofrequency ablation because it preserves the tumor antigens and allows for immune cells to be exposed to a larger amount of intact tumor antigens [6]. In studying patients receiving palliative cryotherapy for dysphagia secondary to esophageal cancer, an improved response rate was observed when combining cryotherapy with chemoradiotherapy, and tissue from patients receiving that combination therapy contained more intratumoral lymphocytes [7]. As it relates to esophageal cancer, one study demonstrated that liquid nitrogen spray cryotherapy provided relief from dysphagia, and 6 of the 9 patients with locally advanced cancer who also completed chemoradiation had no residual tumor cells in their subsequent mucosal biopsies [7].

Cryotherapy alone does not typically create a sufficiently strong immune response to cause cancer regression; however, the immune system’s exposure to a larger number of antigens combined with immunotherapy agents bolsters a stronger tumor-specific immune response [6]. One study demonstrated that combining cryotherapy with immune checkpoint inhibitors revealed increased T lymphocytes in tumor tissue and fewer immunosuppressive cells [8]. It concluded that combining cryotherapy with immune checkpoint inhibitors can relieve the immunosuppressive environment that tumors create while enhancing the T cell response against cancer. Additionally, researchers found that combining cryotherapy and immunotherapy for mice led to reduced tumor growth, low rates of metastases, and increased survival [9]. The treatment had induced anti-tumor memory, which even provided protection against repeated challenges.

Cryotherapy provides both mechanistic destruction of tumors and promotes immune system response against cancer cells, bolstered when combined with immunotherapy.

## 11. Conclusions/Future Directions

Cryotherapy has emerged as a safe and effective endoscopic ablative therapy for neoplastic conditions of the upper gastrointestinal tract, particularly Barrett’s esophagus–associated dysplasia and early carcinoma. It achieves high rates of complete eradication of dysplasia and intestinal metaplasia with a favorable safety profile and minimal serious adverse events [35,39]. In addition to its established role in Barrett’s esophagus, cryotherapy serves as a valuable alternative ablative option for selected patients, including those with RFA-refractory disease or individuals unsuitable for endoscopic resection. Although most existing studies focus on short Barrett’s segments, longer segments may require more extensive ablation and carry an increased risk of stricture formation. Data on long-term outcomes remain limited [35], highlighting the need for standardized treatment protocols to define optimal dosimetry, treatment intervals, and follow-up strategies. Beyond its mechanical ablation properties, cryotherapy’s unique ability to preserve extracellular matrix architecture and promote favorable wound healing distinguishes it from thermal modalities. Furthermore, its immunomodulatory potential—through cryo-immunologic activation—offers promising synergy with systemic and immune checkpoint therapies in advanced or unresectable neoplasia [8,52,53]. As device technology advances and evidence matures, cryotherapy is likely to evolve from a salvage technique to a mainstream therapeutic modality and may ultimately become an integral component of multimodal management for upper gastrointestinal malignancies. We believe funding research in the development of safe cryogens, understanding better mechanisms of injury, immune cryobiology, and delivery systems is key to progress and adoption in GI luminal cryotherapy. Global Gastrointestinal societies and industry collaboration are key to success.

## Data Availability

Since this is a review article, all information and opinions are based on publicly available research in the PubMed database. Deidentified Images are original and provided by the senior author V.J.

## Abbreviations

GEJ (Gastroesophageal Junction), HGD (High Grade Dysplasia), OCT (Optical Coherance Tomography), CBA (Cryoballoon Ablation), CE-HGD (complete eradication of High-grade dysplasia), LNSCT (liquid nitrogen spray cryotherapy), CE-IM (complete eradication of Intestinal Metaplasia), RFA, (radiofrequency ablation).
